# Supplementary material for: Biogenic action of Lactobacillus plantarum SBT2227 promotes sleep in Drosophila melanogaster
Source: iScience. 2022 Jun 17;25(7):104626. doi: 10.1016/j.isci.2022.104626 (PMC9257349; doi:10.1016/j.isci.2022.104626)
Supplement: Document S1. Figures S1–S7 [file mmc1.pdf]

iScience, Volume *25*

Supplemental information

Biogenic action of *Lactobacillus plantarum* SBT2227 promotes sleep in *Drosophila melanogaster*

Taro Ko, Hiroki Murakami, Azusa Kamikouchi, and Hiroshi Ishimoto

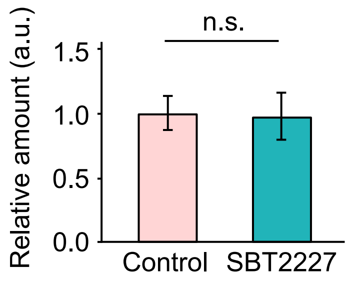

Figure S1. Flies fed the SBT2227 food as well as the control food, related to Figure 1.
The amount of food consumed for 3 h in response to the control food and SBT2227 food was measured. Bars represent mean ± SD. *n* = 5 for control group, *n* = 6 for SBT2227 group Student's *t*-test was applied for statistical analysis. n.s.; not significant. See Table S2 for detailed statistics.

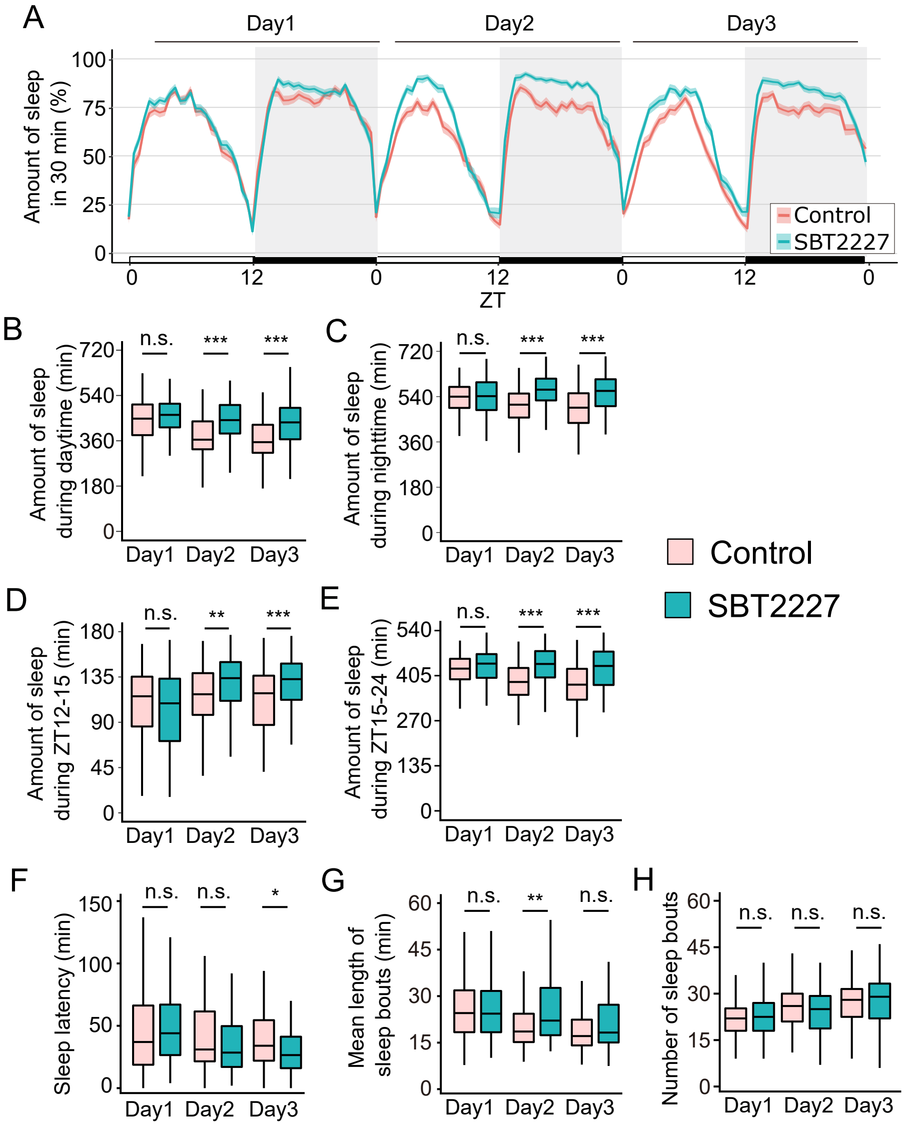

Figure S2. The sleep-promoting effects of SBT2227 were confirmed in another wild-type strain, the genetic background of which differed from that of *Canton-S*^{2202u}, related to Figure 1.

(A) Sleep patterns of female *Amherst-3* fed control food (red) or SBT2227 food (green). Sleep traces are presented as mean ± SEM. (B) Amount of sleep during daytime (ZT0-12), (C) amount of sleep during night-time (ZT12-15), amount of sleep at specific timing; (D) ZT12-15 and (E) ZT15-24, and (F) sleep latency. n = 96 for each group. The Welch's t-test was applied for (B), and the Wilcoxon-Mann-Whitney test was applied for the others. *p*-values were adjusted by Bonferroni correction. * $p < 0.05$, ** $p < 0.01$; *** $p < 0.001$, n.s.; not significant. See Table S2 for detailed statistics.

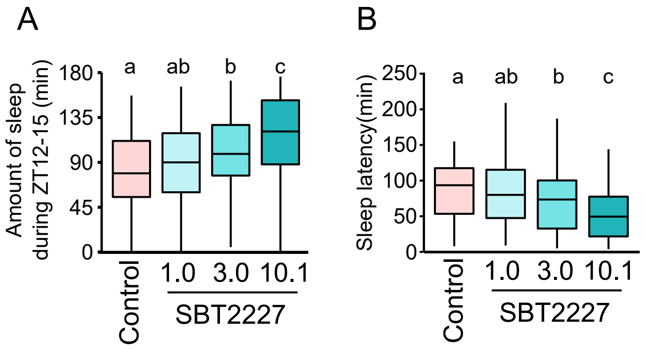

Figure S3. The sleep-promoting effects of SBT2227 are dose-dependent, related to Figure 1.
Flies were treated with different concentrations of SBT2227 (1.01, 3.03, and 10.1 mg/ml). The time period of interest was set at ZT12-15 on day 3. (A) Amount of sleep (Kendall rank correlation coefficient: *tau* = 0.22, p = 8.58e-9). (B) Sleep latency (Kendall rank correlation coefficient: *tau* = -0.22, p = 4.59e-9). The concentration of SBT2227 is shown below each graph. n = 96 in each group. Dwass-Steele-Critchlow-Frigner test was applied for multiple comparisons. Statistical significance was set at $p < 0.05$, and different letters indicate statistical differences between groups. See Table S2 for detailed statistics.

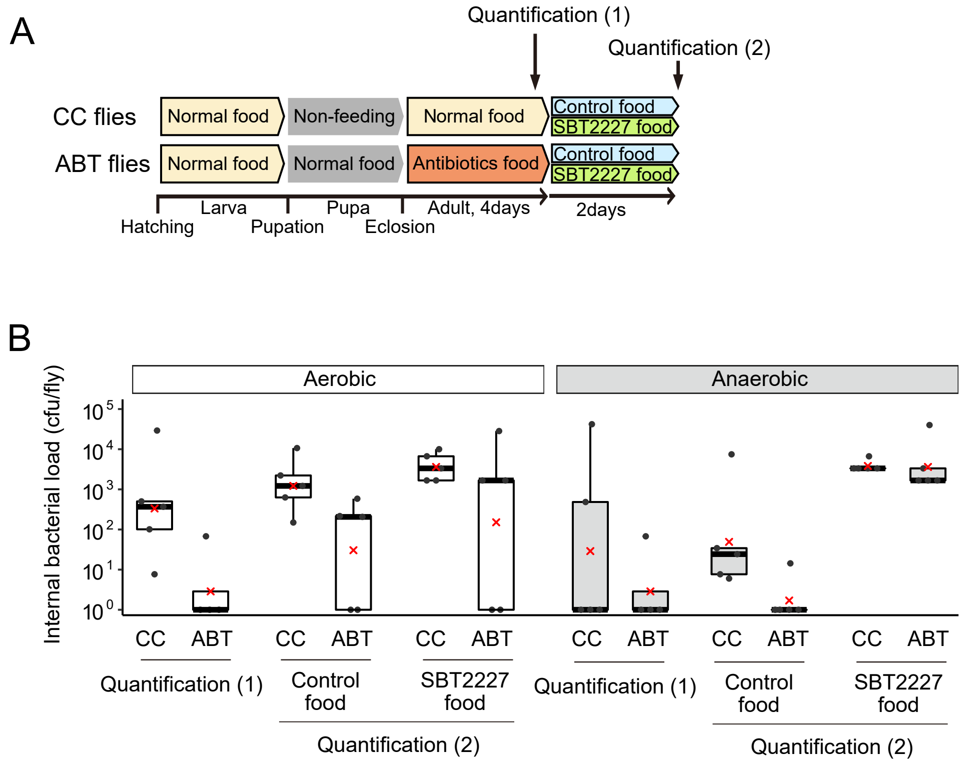

Figure S4. Internal bacteria loads were measured in different food conditions, related to Figure 3. (A) Schematic diagram indicates the experimental flow. (B) The number of viable aerobic or anaerobic bacteria in the gut was determined. *n* = 5 for each measurement. Red crosses indicate average values.

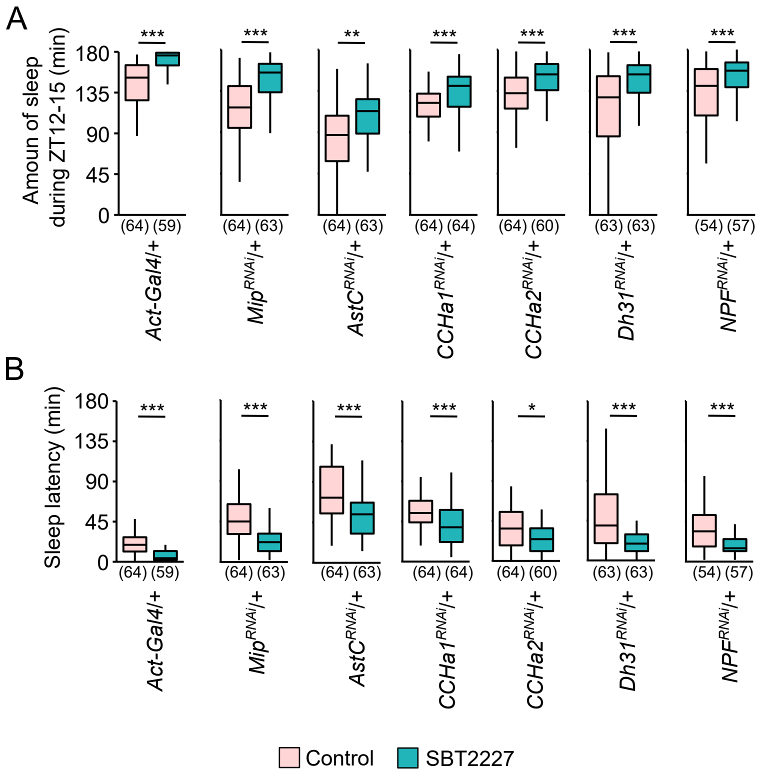

Figure S5. SBT2227 affected sleep in *Gal4* and *UAS* parental strains, related to Figure 7.
(A) Amount of sleep and (B) sleep latency were measured. Sample sizes are indicated below each graph. The Wilcoxon-Mann-Whitney test was applied for statistical analysis in each genotype. * $p < 0.05$, ** $p < 0.01$, *** $p < 0.001$. See Table S2 for detailed statistics.

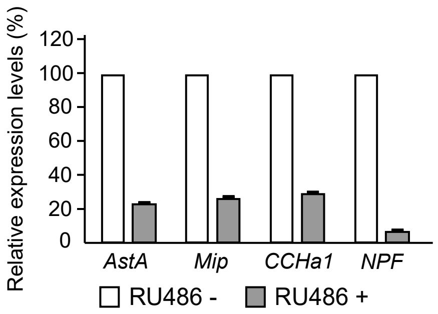

Figure S6. In flies with both *GeneSwitch* and *UAS-RNAi*, the expression of target genes was suppressed by treatment with RU486, related to Figure 7.
Gene expression levels were measured on the second day after administration of RU486. *n* = 3, Bar graphs show mean and standard deviation.

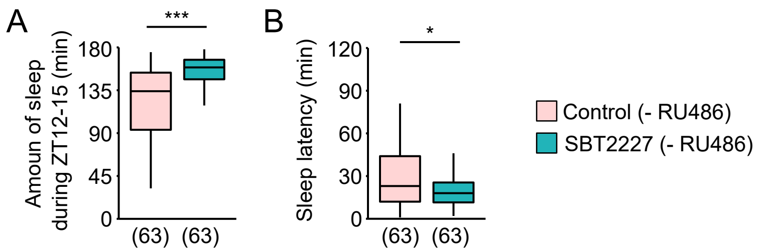

Figure S7. Sleep-promoting effects of SBT2227 were not suppressed in *tub5-GS-Gal4 > NPF^{RNAi}* flies treated with RU486 solvent, related to Figure 7.

(A) Amount of sleep (B) Sleep latency Sample sizes are indicated below each graph. The Wilcoxon-Mann-Whitney test was applied for statistical analysis in each genotype. * $p < 0.05$, *** $p < 0.001$. See Table S2 for detailed statistics.
